# Mathematical Modeling, Analysis, and Simulation of Tumor Dynamics with Drug Interventions

**DOI:** 10.1155/2019/4079298

**Published:** 2019-10-08

**Authors:** Pranav Unni, Padmanabhan Seshaiyer

**Affiliations:** ^1^American International School Chennai, Chennai, Tamilnadu, India; ^2^George Mason University, Fairfax, Virginia, USA

## Abstract

Over the last few decades, there have been significant developments in theoretical, experimental, and clinical approaches to understand the dynamics of cancer cells and their interactions with the immune system. These have led to the development of important methods for cancer therapy including virotherapy, immunotherapy, chemotherapy, targeted drug therapy, and many others. Along with this, there have also been some developments on analytical and computational models to help provide insights into clinical observations. This work develops a new mathematical model that combines important interactions between tumor cells and cells in the immune systems including natural killer cells, dendritic cells, and cytotoxic CD8^+^ T cells combined with drug delivery to these cell sites. These interactions are described via a system of ordinary differential equations that are solved numerically. A stability analysis of this model is also performed to determine conditions for tumor-free equilibrium to be stable. We also study the influence of proliferation rates and drug interventions in the dynamics of all the cells involved. Another contribution is the development of a novel parameter estimation methodology to determine optimal parameters in the model that can reproduce a given dataset. Our results seem to suggest that the model employed is a robust candidate for studying the dynamics of tumor cells and it helps to provide the dynamic interactions between the tumor cells, immune system, and drug-response systems.

## 1. Introduction

Cancer is one of the leading causes of death in the world today. By 2030, over 13 million are estimated to harbor some form of the disease. While there have been many developments in cancer therapies including surgery, chemotherapy, immunotherapy, and radiotherapy, there is still a lot that is unknown about the dynamics of how cancer cells are created, propagated, and destroyed.

Over the past few decades, there have been several experimental approaches and interventions developed that have helped us to understand the dynamics of tumor growth and its interactions with the immune system [1, 2]. This has also helped to inform how specific interventions such as immunotherapy can help strengthen our own ability to fight cancer by improving the effectiveness of the immune system [3–5]. While these developments have helped enhance our understanding about cancer dynamics, there are still several challenges in these experimental approaches to fully understand the interactions with the immune system.

In the last two decades, there have also been several experimental advances in developing interventional therapies for cancer such as immunotherapy, virotherapy, targeted drug therapies, and chemotherapy. Along with these experimental developments, there have been some advances in scientific and engineering solutions to capture the dynamics of cancer. One of the promising approaches includes mathematical modeling [6, 7], which involves identifying the cells that play a role in cancer propagation, interactions between these bodies, and description of the dynamics of this interaction that has helped estimate parameters, perform stability analysis, and predict tumor dynamics [8–13]. These models have been able to demonstrate the importance of the presence of immune components for explaining clinically observed phenomena such as tumor dormancy [14], tumor size oscillations and regressions [15–18], nonspatial models of tumor and immune system interactions [19, 20], and tumor growth coupled with immunotherapy [8, 9, 12, 13, 21, 22].

These mathematical models are often coupled system of governing differential equations that describe the dynamics of each of the interacting component cells. Specifically, the interactions between tumor growth and the immune system are often described using a system of coupled differential equations with prescribed initial conditions. These equations include nonlinear interactions and do not often admit an exact solution and therefore require computational methods to solve them. While these mathematical models have provided useful information regarding the importance of the immune system in controlling tumor growth, there is still a great need to continue to enhance existing models to incorporate new clinical developments and biological discoveries. For example, there have been studies suggesting the effectiveness of chemotherapy with immunotherapy and vaccine therapies [1, 13, 23]. The focus of this paper is to enhance existing models of tumor growth that incorporate tumor dynamics in conjunction with the immune system response and also study the effect of additional interventions including antitumor vaccination and immunotherapies along with chemotherapy.

Of the many clinical approaches that are tested for cancer therapy, one of the popular approaches includes drug therapy to the tumor microenvironment. To understand the impact of the drugs delivered to the tumor cell site, it is important to include the effect of these drugs into the models as well. Towards this end, we develop a mathematical model that will combine essential interactions between growing tumor cells and cells of the innate and specific immune system coupled with models for drug delivery to these cell sites. Our goal is to use these models developed to study the effectiveness of anticancer drugs to reduce tumor growth.

The growth of tumors has also been attributed to the dynamics of the cellular immune system within the human host. Two principal components of this immune system include the natural killer cells and cytotoxic CD8^+^ T cells which are known to kill tumor cells. Besides these, other important antigen-presenting cells include the dendritic cells that help stimulate, recruit, and activate the immune system. While research has been growing to discover potential mechanisms to describe immune system interactions with growing tumors, there is enough evidence that the dynamics of natural kills cells, cytotoxic CD8^+^ T-cells, and dendritic cells influence tumor dynamics. The model presented in this work will account for the influence of these as well.

Over the years, there has been a lot of development in mathematical modeling of cancer. However, the mechanisms that are involved in the interactions of tumor cells with the immune system are still not clear. This paper attempts to make a new contribution in this direction by developing a coupled mathematical model that incorporates tumor dynamics and interactions between the dendritic cells, natural killer cells, and CD8^+^ T cells. Additionally, the model incorporates and studies the influence of various drug therapies including immunotherapy and chemotherapy. Finally, a new parameter estimation technique is proposed that helps to estimate parameters optimally for a given extrapolated dataset.

## 2. Models and Background

In this work, we will consider a model that consists of four main cell populations including tumor cells (*T*(*t*)), natural killer cells (*N*(*t*)), dendritic cells (*D*(*t*)), and cytotoxic CD8^+^ T cells denoted by (*L*(*t*)). The dynamics of these cells will include interactions between each other as well as dynamics generated by interaction with chemotherapy as well immunotherapy drug concentrations in the blood stream.

For developing the model for each of the cell populations, a standard approach is to begin with applying conservation of mass with diffusion and activation. This would often yield the following equation for the dynamics of the various types of cells:

(1) $$\begin{array}{c} \frac{\partial \left[\cdot \right]}{\partial t}+\nabla \cdot \left(\vec{u}\left[\cdot \right]\right)-\delta_{D}\nabla^{2}\left[\cdot \right]=f\left(\cdot \right)-g\left(\cdot \right)-K_{\left[\cdot \right]}z\left(M\right)\left[\cdot \right]. \end{array}$$

Here, the functions *f*(·) and *g*(·) will be based on proliferation rates, competition terms, and inhibition terms based on the respective roles of each type of cell.

Also, note that, in all the models, we will consider the effect of a chemotherapy drug (dynamics described later) kill term through *K*_[·]_*z*(*M*)[·]. The term *z*(*M*)=1 − *e*^−*M*^ is used to denote the fact that chemotherapeutic drugs (for example, doxorubicin) are only effective during certain phases of the cell cycle and pharmacokinetics. The values of the kill parameters *K*_[·]_ for the four cell population considered here are based on their ability to disrupt the process of division and growth [24, 25]. Note that if *K*_[·]_=0, the equation is not impacted by the drug kill term.

While equation (1) includes both the diffusion term and the advection term due to blood velocity $\vec{u}$, we will consider only the temporal dynamics in this work and hence the associated ordinary differential equation:

(2) $$\begin{array}{c} \frac{\text{d}\left[\cdot \right]}{\text{d}t}=f\left(\cdot \right)-g\left(\cdot \right)-K_{\left[\cdot \right]}z\left(M\right)\left[\cdot \right]. \end{array}$$

### 2.1. Modeling Tumor Cells

We begin with modeling tumor cells *T* which are assumed to have a proliferation rate that can be modeled by a logistic growth law *aT*(1 − *bT*), with parameters *a* and *b* denoting the per capita growth rates and reciprocal carrying capacities of the tumor cells [8, 9, 26]. Also, it is known that the growth of the tumor cells is impacted by three different competitive interactions including interactions between tumor cells and dendritic cells, interactions between tumor cells and natural killer cells, and interactions between tumor cells and CD8^+^ T cells [26–28]. Denoting the corresponding competition rates as $\underline{j},c_{1},k$, respectively, introduces the competition term as −(*c*_1_*N*+*j* *D*+*kL*)*T*. The dynamics of tumor cells can then be described by the following ordinary differential equation:

(3) $$\begin{array}{c} \frac{\text{d}T}{\text{d}t}=aT\left(1-bT\right)-\left(c_{1}N+j\;D+kL\right)T-K_{T}z\left(M\right)T. \end{array}$$

### 2.2. Modeling Natural Killer Cells

To model natural killer (NK) cells, we will assume that these cells have a constant source *s*_1_ as well as a NK cell recruitment term that can be represented through a modified Michaelis–Menten term (commonly used to govern cell-cell interactions):

(4) $$\begin{array}{c} g_{1}\cdot \frac{T^{2}}{h_{1}+T^{2}}\cdot N, \end{array}$$

where *g*_1_ denotes the maximum NK cell recruitment rate by tumor cells and *h*_1_ denotes the steepness coefficient of the NK cell recruitment curve [8].

Next, the growth of NK cells will be impacted by two different interactions, namely, the interaction between NK cells and tumor cells [29] and the interaction between NK cells and dendritic cells [30–33]. We also introduce parameters *c*_2_, *d*_1_ to be the rates of killing (due to tumor cells) and proliferation (due to dendritic cells) of NK cells, respectively. The governing differential equation for the dynamics of NK cells then can be described as

(5) $$\begin{array}{c} \frac{\text{d}N}{\text{d}t}=s_{1}+\frac{g_{1}NT^{2}}{h_{1}+T^{2}}-\left(c_{2}T-d_{1}D\right)N-K_{N}z\left(M\right)N-eN. \end{array}$$

Note that we have also included a natural death of NK cells through −*eN*.

### 2.3. Modeling Dendritic Cells

Dendritic cells play an important role in the immune system response and in controlling tumor growth. Also known as antigen-presenting cells, they update and present antigens to CD8^+^ T cells. Some of the earlier models [8, 13] in the literature have not incorporated the dynamics of dendritic cells in directly suppressing tumor growth, stimulating resting NK cells, and impacting the dynamics of CD8^+^ T cells. There is, however, experimental evidence that dendritic cells play an important role in modeling tumor immunotherapy [28].

To study the dynamics of dendritic cells, we will assume *s*_2_ to be a constant source of dendritic cells, *d*_2_ to be the rate at which NK cells kill dendritic cells, *d*_3_ to be a proliferation rate of dendritic cells due to tumor cells, *f*_1_ to be the rate corresponding to the interaction of dendritic cells with CD8^+^ T cells, and *g* to be the natural death rate of dendritic cells. We then have

(6) $$\begin{array}{c} \frac{\text{d}D}{\text{d}t}=s_{2}-\left(f_{1}L+d_{2}N-d_{3}T\right)D-K_{D}z\left(M\right)D-gD. \end{array}$$

### 2.4. Modeling Cytotoxic CD8^+^ T Cells

Among many factors that impact the growth of tumor cells, it is well known that CD8^+^ T cells are an important component of the immune system that kills tumor cells. It has been seen that active tumor-specific CD8^+^ T cells are only present in large numbers when tumor cells are present [16, 34], and after some interactions with tumor cells, they become inactive [29]. It has been observed that mature CD8^+^ T cells can remove dendritic cells [35, 36].

In our model, to describe the dynamics of the CD8^+^ T-cells, we will consider *f*_2_ to be the rate of interaction between dendritic cells and tumor cells to activate CD8^+^ T cells; −*hLT* denotes the form of competition between CD8^+^ T cells and tumor cells, and −*iL* denotes the natural death rate of CD8^+^ T cells.

It has also been seen that CD8^+^ T cells may be recruited by the debris from tumor cells lysed by NK cells [37]. To include this effect, we will incorporate in our model a recruitment term that is proportional to the number of cells killed, which is denoted as *r*_1_*NT*. We will also need an additional term that helps describe the regulation and suppression of CD8^+^ T-cell activity when there are high levels of activated CD8^+^ T cells without responsiveness to cytokines in the system [38, 39]. This term is denoted by *uNL*^2^. We also include CD8^+^ T activation by IL-2 immunotherapy which is in the form of a drug that influences the immune system's efficacy and described via a Michaelis–Menten interaction term [16]:

(7) $$\begin{array}{c} \frac{p_{I}LI}{g_{I}+I}. \end{array}$$

Here, *I* refers to the immunotherapy drug concentration in the bloodstream. The model for the dynamics for the CD8^+^ T cell growth population becomes

(8) $$\begin{array}{c} \frac{\text{d}L}{\text{d}t}=f_{2}DT-hLT-uNL^{2}+r_{1}NT+\frac{p_{I}LI}{g_{I}+I}-K_{L}z\left(M\right)L-iL. \end{array}$$

### 2.5. Modeling Drug and Vaccine Interventions

In this study, we incorporate a variety of external intervention treatment options including tumor-infiltrating lymphocyte (TIL) injections as well as chemotherapy and immunotherapy drugs. TIL drug intervention may be thought of as an immunotherapy approach in which the CD8^+^ T-cells are promoted through antigen-specific cytolytic immune cells. We do this by adding the term *v*_*L*_=*v*_*L*_(*t*) in equation (8) and we have

(9) $$\begin{array}{c} \frac{\text{d}L}{\text{d}t}=f_{2}DT-hLT-uNL^{2}+r_{1}NT+\frac{p_{I}LI}{g_{I}+I}-K_{L}z\left(M\right)L-iL+v_{L}. \end{array}$$

To include the chemotherapy and immunotherapy drugs, we describe the dynamics of the respective concentrations in the blood stream as follows:

(10) $$\begin{array}{c} \frac{\text{d}M}{\text{d}t}=v_{M}\left(t\right)-d_{4}M,\\ \frac{\text{d}I}{\text{d}t}=v_{I}\left(t\right)-d_{5}I. \end{array}$$

The drug intervention terms in these equations reflect the amount of chemotherapy and immunotherapy drug given over time. Note that we assume that the chemotherapy and immunotherapy drugs will be eliminated from the body over time at a rate proportional to its concentration, and these are given by *d*_4_*M* and *d*_5_*I*, respectively.

### 2.6. Overall Model

From Sections 2.1–2.5, we have the following overall model:

(11) $$\begin{array}{c} \dot{T}=aT\left(1-bT\right)-\left(c_{1}N+jD+kL\right)T-K_{T}z\left(M\right)T,\\ \dot{N}=s_{1}+\frac{g_{1}NT^{2}}{h_{1}+T^{2}}-\left(c_{2}T-d_{1}D\right)N-K_{N}z\left(M\right)N-eN,\\ \dot{D}=s_{2}-\left(f_{1}L+d_{2}N-d_{3}T\right)D-K_{D}z\left(M\right)D-gD,\\ \dot{L}=f_{2}DT-hLT-uNL^{2}+r_{1}NT+\frac{p_{I}LI}{g_{I}+I}-K_{L}z\left(M\right)L-iL+v_{L}\left(t\right),\\ \dot{M}=v_{M}\left(t\right)-d_{4}M,\\ \dot{I}=v_{I}\left(t\right)-d_{5}I. \end{array}$$

**Figure 1:**
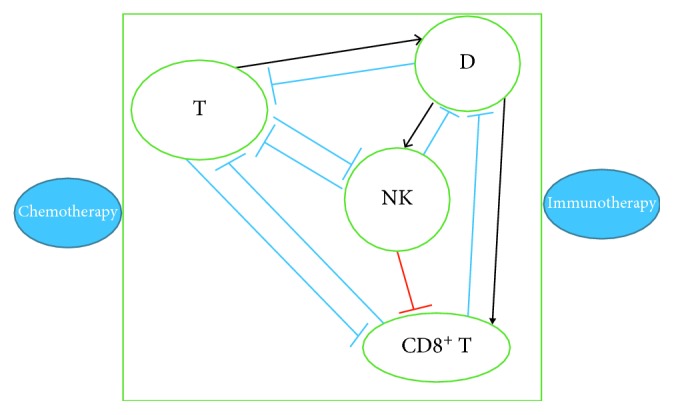
Network of the dynamics for system (11).

Figure 1 illustrates the network of the dynamics for system (11). Sharp arrows represent reproduction or activation, and blocked arrows represent inhibition or killing. The blocked arrow in red represents the nonlinear interaction. Note that the suppressing effects of the drug are not shown explicitly in the figure but included in the model.

## 3. Stability Analysis

In this section, we employ mathematical analysis to identify conditions that can help eliminate tumor cells. Also, we will determine conditions for when tumor-free equilibrium is unstable and the tumor grows without bound.

We now consider the system of (11) in the absence of treatment. When we eliminate chemotherapy and immunotherapy, the system reduces to a four-population system of ODEs. Let *E*(*T*^*∗*^, *N*^*∗*^, *D*^*∗*^, *L*^*∗*^) be an equilibrium point of the system described by the system without drug intervention.

At an equilibrium point, we have

(12) $$\begin{array}{c} \frac{\text{d}T}{\text{d}t}=\frac{\text{d}N}{\text{d}t}=\frac{\text{d}D}{\text{d}t}=\frac{\text{d}L}{\text{d}t}=0. \end{array}$$

Since we assume there is constant recruitment through source terms *s*_1_ and *s*_2_, both not equal to zero, there is no trivial equilibrium which implies

(13) $$\begin{array}{c} E\left(T^{*},N^{*},D^{*},L^{*}\right)\neq \left(0,0,0,0\right). \end{array}$$

For tumor-free equilibrium, at an equilibrium point, we have d*N*/d*t*=0 which yields

(14) $$\begin{array}{c} s_{1}+d_{1}D^{*}N^{*}-eN^{*}=0, \end{array}$$

which yields

(15) $$\begin{array}{c} N^{*}=\frac{s_{1}}{e-d_{1}D^{*}}. \end{array}$$

Similarly, setting d*D*/d*t*=0 at an equilibrium point yields

(16) $$\begin{array}{c} s_{2}-d_{2}N^{*}D^{*}-gD^{*}=0. \end{array}$$

Substituting (15) in (16), we get

(17) $$\begin{array}{c} gd_{1}D^{*2}-\left(s_{2}d_{1}+d_{2}s_{1}+eg\right)D^{*}+es_{2}=0. \end{array}$$

Solving the quadratic equation yields

(18) $$\begin{array}{c} D_{1,2}^{*}=\frac{\left(d_{1}s_{2}+d_{2}s_{1}+eg\right)\pm \sqrt{{\left(d_{1}s_{2}+d_{2}s_{1}+eg\right)}^{2}-4ges_{2}}}{2gd_{1}}. \end{array}$$

Hence, we have 2 tumor-free equilibrium given by *E*_1_(0, *N*^*∗*^, *D*_1_^*∗*^, 0) and *E*_2_(0, *N*^*∗*^, *D*_2_^*∗*^, 0).

For these to have biological meaning, we need

(19) $$\begin{array}{c} e-d_{1}D^{*}>0, \end{array}$$

(20) $$\begin{array}{c} d_{1}s_{2}+d_{2}s_{1}+eg\geq 2\sqrt{ges_{2}}. \end{array}$$

These conditions suggest critical values for the death rate and the source term for NK cells to be

(21) $$\begin{array}{c} e=d_{1}D^{*},\\ s_{1}=\frac{2\sqrt{ges_{2}}-\left(d_{1}s_{2}+eg\right)}{d_{2}}, \end{array}$$

in order for the tumor-free equilibrium point to be positive for biological significance.

The Jacobian matrix for linearization of system (11) without drug intervention is given by

(22) $$\begin{array}{c} \left[\begin{array}{cccc} a_{11} & a_{12} & a_{13} & a_{14}\\ a_{21} & a_{22} & a_{23} & a_{24}\\ a_{31} & a_{32} & a_{33} & a_{34}\\ a_{41} & a_{42} & a_{43} & a_{44} \end{array}\right], \end{array}$$

where

(23) $$\begin{array}{c} a_{11}=a-2abT^{*}-c_{1}N^{*}-jD^{*}-kL^{*},\\ a_{12}=-c_{1}T^{*},\\ a_{13}=-jT^{*},\\ a_{14}=-kT^{*},\\ a_{21}=\frac{2g_{1}N^{*}h_{1}T^{*}}{{\left(h_{1}+T\right)}^{2}}-c_{2}N^{*},\\ a_{22}=-c_{2}T^{*}+d_{1}D^{*}-e,\\ a_{23}=d_{1}N^{*},\\ a_{24}=0,\\ a_{31}=d_{3}D^{*},\\ a_{32}=-d_{2}D^{*},\\ a_{33}=-\left(f_{1}L^{*}+d_{2}N^{*}-d_{3}T^{*}+g\right),\\ a_{34}=-f_{1}D^{*},\\ a_{41}=f_{2}D^{*}-hL^{*}+r_{1}N^{*},\\ a_{42}=-uL^{{*}^{2}}+r_{1}T^{*},\\ a_{43}=f_{2}T^{*},\\ a_{44}=-\left(hT^{*}+2uN^{*}L^{*}+i\right). \end{array}$$

Evaluating these terms at the general tumor-free equilibrium point gives

(24) $$\begin{array}{c} A=\left[\begin{array}{cccc} a-c_{1}N^{*}-jD^{*} & 0 & 0 & 0\\ \frac{2g_{1}N^{*}h_{1}T^{*}}{{\left(h_{1}+T\right)}^{2}}-c_{2}N^{*} & d_{1}D^{*}-e & d_{1}N^{*} & 0\\ d_{3}D^{*} & -d_{2}D^{*} & -\left(d_{2}N^{*}+g\right) & -f_{1}D^{*}\\ f_{2}D^{*}+r_{1}N^{*} & 0 & 0 & -i \end{array}\right]. \end{array}$$

To solve for the eigenvalues *λ*, we solve the equation det(*A* − *λI*)=0 or

(25) $$\begin{array}{c} \det\left[\begin{array}{cccc} a-c_{1}N^{*}-jD^{*}-\lambda & 0 & 0 & 0\\ \frac{2g_{1}N^{*}h_{1}T^{*}}{{\left(h_{1}+T\right)}^{2}}-c_{2}N^{*} & d_{1}D^{*}-e-\lambda & d_{1}N^{*} & 0\\ d_{3}D^{*} & -d_{2}D^{*} & -\left(d_{2}N^{*}+g\right)-\lambda & -f_{1}D^{*}\\ f_{2}D^{*}+r_{1}N^{*} & 0 & 0 & -i-\lambda \end{array}\right]=0, \end{array}$$

which yields

(26) $$\begin{array}{c} \left(a-c_{1}N^{*}-jD^{*}-\lambda \right)\left(-i-\lambda \right)\det\left(B-\lambda I\right)=0, \end{array}$$

where matrix *B* is given by

(27) $$\begin{array}{c} B=\left[\begin{array}{cc} d_{1}D^{*}-e & d_{1}N^{*}\\ -d_{2}D^{*} & -\left(d_{2}N^{*}+g\right) \end{array}\right]. \end{array}$$

It may be noted that *trace* and *determinant* for matrix *B* can be computed to be

(28) $$\begin{array}{c} \text{tr}\left(B\right)=\left(d_{1}D^{*}-e\right)-\left(d_{2}N^{*}+g\right), \end{array}$$

(29) $$\begin{array}{c} \det\left(B\right)=d_{1}d_{2}N^{*}D^{*}-\left(d_{1}D^{*}-e\right)\left(d_{2}N^{*}+g\right). \end{array}$$

Solving (26), we compute the eigenvalues to be

(30) $$\begin{array}{c} \lambda_{1}=a-c_{1}N^{*}-jD^{*},\\ \lambda_{2}=-i, \end{array}$$

and *λ*_3_ and *λ*_4_ to be the roots of the equation:

(31) $$\begin{array}{c} \lambda^{2}-\lambda \left[\left(d_{1}D^{*}-e\right)-\left(d_{2}N^{*}+g\right)\right]+d_{1}d_{2}N^{*}D^{*}-\left(d_{1}D^{*}-e\right)\left(d_{2}N^{*}+g\right)=0. \end{array}$$

From (28) and (29), this can be rewritten as

(32) $$\begin{array}{c} \lambda^{2}-\text{tr}\left(B\right)\lambda +\det\left(B\right)=0. \end{array}$$

Using (19) in equations (28) and (29), we can conclude that

(33) $$\begin{array}{c} \text{tr}\left(B\right)<0,\\ \det\left(B\right)>0. \end{array}$$

This implies that the eigenvalues *λ*_3_ and *λ*_4_ that are the roots of equation (32) have negative real parts.

In order for the tumor-free equilibrium to be stable, we require *λ*_1_ < 0, which implies that if the tumor growth rate *a* is lesser than the critical value given by *c*_1_*N*^*∗*^+*JD*^*∗*^, then the tumor population can be eliminated.

## 4. Computational Experiments

In this section, we will consider the system of (11) which will be solved via the Runge–Kutta methods. The parameter values, their units, and their estimated value are itemized next, which we use in our computations.

- *a*: tumor growth rate estimated as 4.31*∗*10^−1^ day^−1^ [9]
- *b*: *b*^−1^ tumor-carrying capacity estimated as 2.17*∗*10^−8^ cells^−1^ [9]
- *c*_1_: NK cell tumor cell kill rate estimated as 3.5 *∗* 10^−6^ cells^−1^ [12]
- *c*_2_: NK cell inactivation rate by tumor cells estimated as 1.0 *∗* 10^−7^ cells^−1^ day^−1^ [9]
- *d*_1_: rate of dendritic cell priming NK cells estimated as 1.0 *∗* 10^−6^ cells^−1^ [12]
- *d*_2_: NK cell dendritic cell kill rate estimated as 4.0 *∗* 10^−6^ cells^−1^ [12]
- *d*_3_: rate of tumor cells priming dendritic cells estimated as 1.0 *∗* 10^−4^*Estimate*
- *e*: death rate of NK cell estimated as 4.12 *∗* 10^−2^ day^−1^ [12]
- *f*_1_: CD8^+^ T cell dendritic cells kill rate estimated as 1.0 *∗* 10^−8^ cells^−1^ [12]
- *f*_2_: rate of dendritic cells priming CD8^+^ T cell estimated as 0.01 cells^−1^ [12]
- *g*: death rate of dendritic cells estimated as 2.4 *∗* 10^−2^ cells^−1^ [12]
- *h*: CD8^+^ T inactivation rate by tumor cells estimated as 3.42 *∗* 10^−10^ cells^−1^ day^−1^ [12]
- *i*: death rate of CD8^+^ T cells estimated as 2.0 *∗* 10^−2^ day^−1^ [9]
- *j*: dendritic cell tumor cell kill rate estimated as 1.0 *∗* 10^−7^ cells^−1^ [12]
- *k*: NK cell tumor cell kill rate estimated as 1.0 *∗* 10^−7^ cells^−1^ [12]
- *s*_1_: source of NK cells estimated as 1.3 *∗* 10^4^ cells^−1^ [12]
- *s*_2_: source of dendritic cell estimated as 4.8 *∗* 10^2^ cells^−1^ [12]
- *u*: regulatory function by Nk cells of CD8^+^ T cells estimated as 1.80 *∗* 10^−8^ cell^−2^ day^−1^ [9]

**Figure 2:**
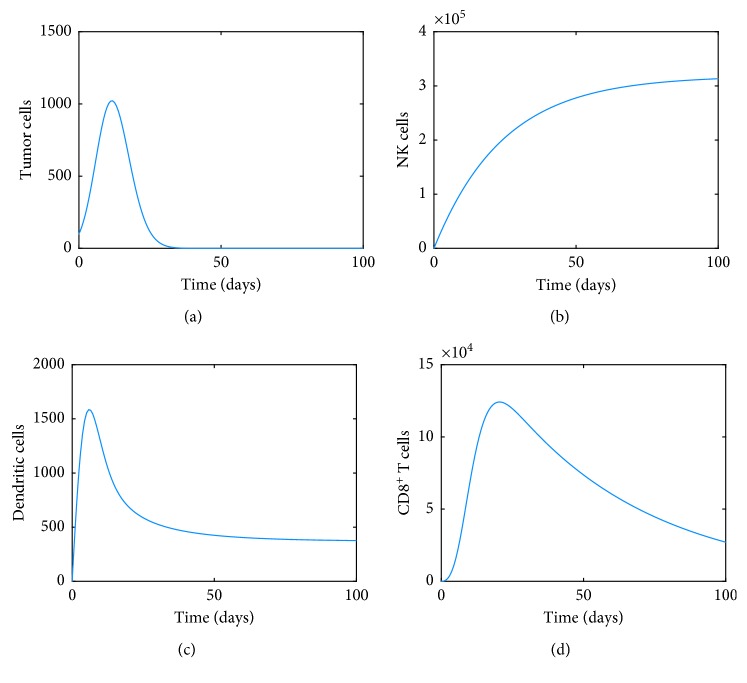
Dynamics of (a) tumor, (b) NK, (c) dendritic, and (d) CD8^+^ T cells.

For the first computation, we will assume there is no additional recruitment terms for CD8^+^ T cells and NK cells (*r*_1_=0, *g*_1_=0, *h*_1_=0), removing some of the regulation, suppression. and activation of CD8^+^ T cells (*p*_*I*_=*g*_*I*_=*u*=0), not including the influence of drug kill terms (*K*_*T*_=*K*_*N*_=*K*_*D*_=*K*_*L*_=0), no influence of drug and vaccine interventions (*v*_*L*_=*v*_*M*_=*v*_*I*_=0) along with the corresponding death rates (*d*_4_=*d*_5_=0). We will also assume *d*_3_=0 which corresponds to the new term that has been added to the model to indicate the growth of dendritic cells being impacted by tumor cells. We will also assume for simplicity and illustration purposes of a weak immune system that the dynamics start with 100 tumor cells with one natural killer, one dendritic, and one CD8^+^ T cell.  Figure 2 shows the dynamics of each of these cells. The tumor cells initially increase to a peak before a full immune clearance starts.

**Figure 3:**
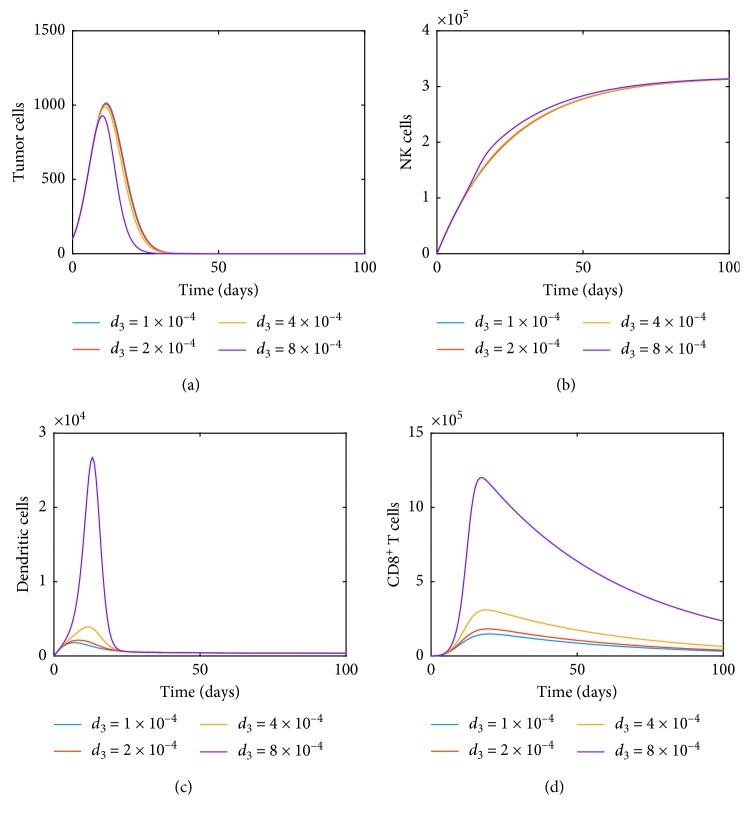
Dynamics of (a) tumor, (b) NK, (c) dendritic, and (d) CD8^+^ T cells as the proliferation rate *d*_3_ is doubled.

Next, we consider the effect of one of the terms in system (11) corresponding to the dynamics of dendritic cells. This is the term *d*_3_*TD* which includes the influence of tumor growth on the dynamics of dendritic cells that all the previous studies have not considered.  Figure 3 illustrates how not only the dendritic cells are impacted but the CD8^+^ T cell dynamics also changes as the proliferation rate *d*_3_ is doubled. For the rest of the simulations, we will include the effect of *d*_3_ and assume the value to be 1 × 10^−4^.

**Figure 4:**
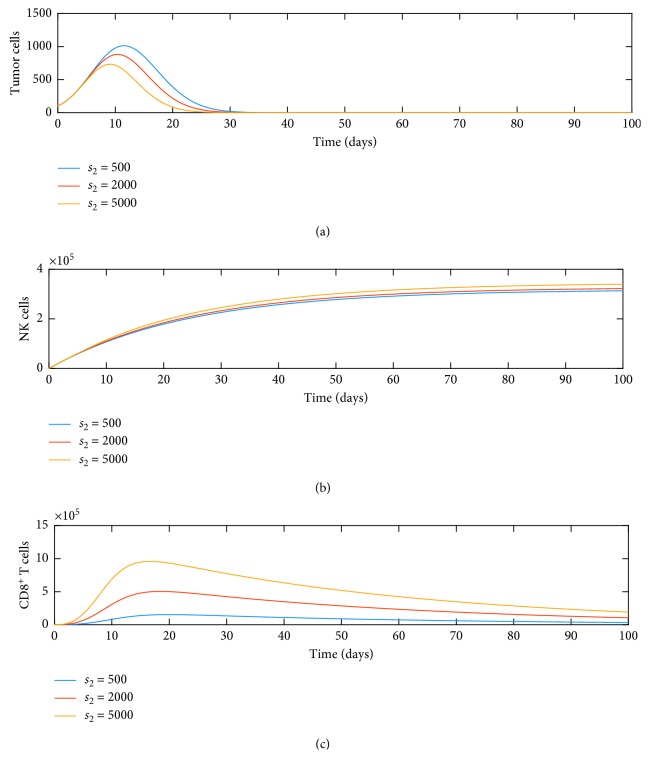
Dynamics of (a) tumor, (b) NK, and (c) CD8^+^ T cells as function of source *s*_2_.

Along with *d*_3_, we also wanted to study the influence of the source term *s*_2_ on the dynamics of tumor, NK, and CD8^+^ T cells.  Figure 4 illustrates this behavior. When we increase the source term of dendritic cells, it increases NK cells and CD8^+^ T cells. We also note that these have an effect on tumor growth as both NK and CD8^+^ T cells can lyse tumor cells. This decrease is also seen in this figure. This suggests that an external source term of dendritic cells has the potential to decrease tumor growth. We also note that dendritic cells play an important role in recruiting CD8^+^ T cells earlier in the tumor growth phase.

**Figure 5:**
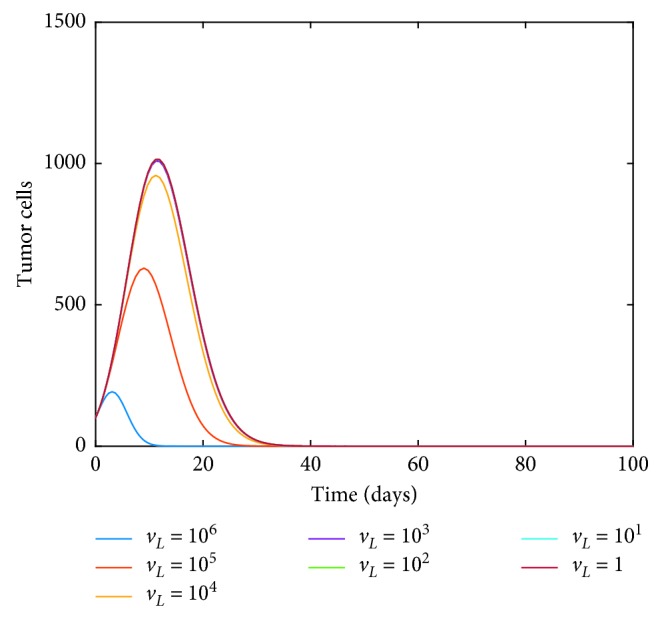
Dynamics of tumor cells as the immunotherapy TIL drug intervention term *v*_*L*_ is varied.

Next, to study the effect of TIL drug intervention term only for the CD8^+^ T-cell population as an immunotherapy where the immune cell levels are boosted by the addition of antigen-specific cytolytic immune cells, we increase the value of *v*_*L*_ from 1 to 10^6^. The result is shown in Figure 5, and we notice that the effect of adding the drug in small doses does not have a big impact on tumor growth.

**Figure 6:**
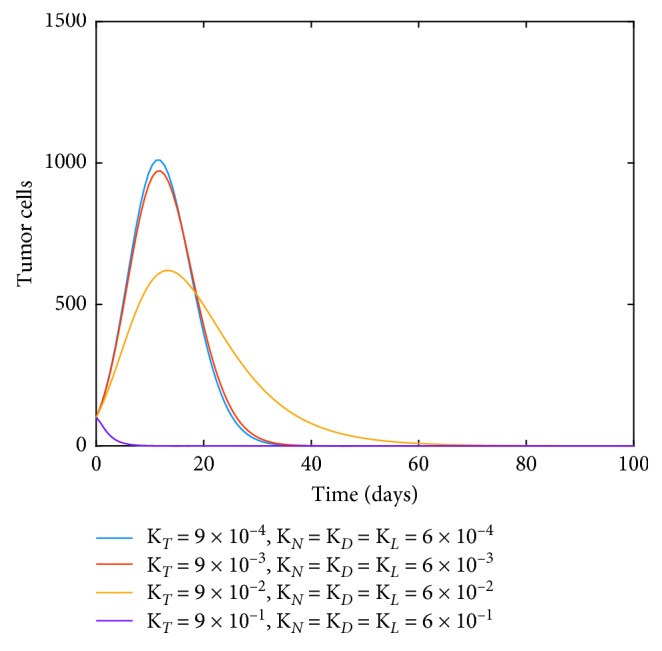
Dynamics of tumor cells as immunotherapy drug intervention with constant *v*_*M*_ and varying levels of chemotherapy drug kill terms.

Next, we consider the effect of the chemotherapy drug only that is introduced through the term *v*_*M*_. We set *v*_*M*_=1 and study the influence of increasing *K*_*T*_ in the dynamics of tumor cells.  Figure 6 illustrates how tumor cells can be reduced through this technique.

We want to point out that we also performed the study on the influence of immunotherapy drug intervention *v*_*I*_ but noticed that even with inclusion of a CD8^+^ T activation described via Michaelis–Menten interaction given by

(34) $$\begin{array}{c} \frac{p_{I}LI}{g_{I}+I}, \end{array}$$

there was negligible effect on the dynamics of all four cells. We also noted that there was not much effect in the dynamics of NK cells through the recruitment terms involving *g*_1_ and *r*_1_.

**Figure 7:**
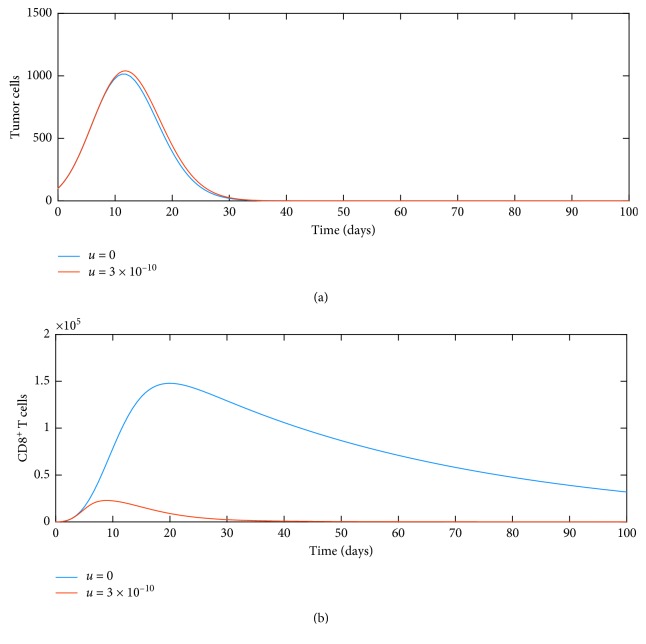
Influence of nonlinearity on dynamics of (a) tumor and (b) CD8^+^ T cells.

Next, we turn our attention to the effect of the nonlinear term introduced as an inactivation term, which describes the regulation and suppression of CD8^+^ T-cell activity [38, 39]. Specifically, Figure 7 shows the effect of the nonlinear term in system (11) for parameters *u*=0 and *u*=3 × 10^−10^, and the dynamics show that there is a drastic drop in the number of cytotoxic CD8^+^ T cells while a small increase in tumor cell growth.

In summary, our computations seem to suggest that a combination of immunotherapy through TIL drug intervention *v*_*M*_ along with chemotherapy through *v*_*L*_ provides an optimal way to reduce tumor growth. Taking this into account and removing terms that had negligible effects, one can consider the following simplified system that captures most prominent features:

(35) $$\begin{array}{c} \dot{T}=aT\left(1-bT\right)-\left(c_{1}N+jD+kL\right)T-K_{T}z\left(M\right)T,\\ \dot{N}=s_{1}-\left(c_{2}T-d_{1}D\right)N-K_{N}z\left(M\right)N-eN,\\ \dot{D}=s_{2}-\left(f_{1}L+d_{2}N-d_{3}T\right)D-K_{D}z\left(M\right)D-gD,\\ \dot{L}=f_{2}DT-hLT-uNL^{2}-K_{L}z\left(M\right)L-iL+v_{L}\left(t\right),\\ \dot{M}=v_{M}\left(t\right)-d_{4}M. \end{array}$$

**Figure 8:**
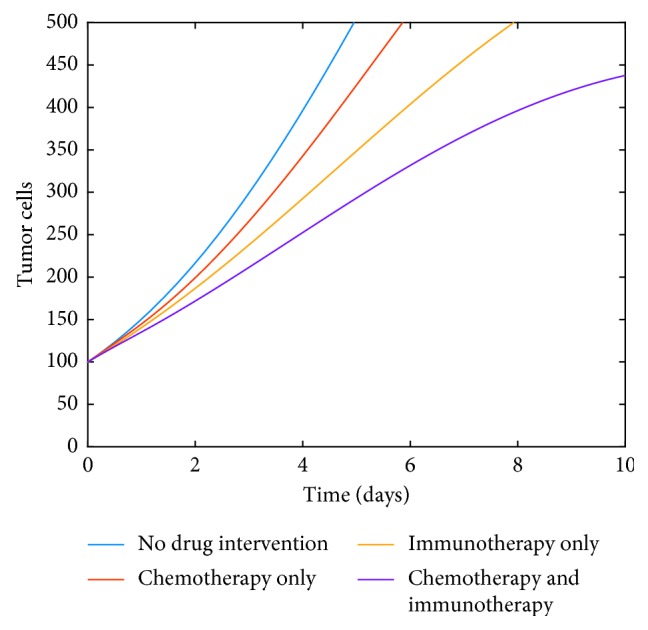
Influence of no drug intervention, independent drug interventions, and combined drug interventions.

Figure 8 clearly shows that combined chemotherapy and immunotherapy drug intervention helps reduce the tumor growth for system (35). We have used *K*_*T*_=9 × 10^−2^, *K*_*D*_=*K*_*N*_=*K*_*L*_=6 × 10^−2^ with *v*_*L*_=10^6^ and *v*_*M*_=1 for our computations.

## 5. Parameter Estimation

In this section, we focus on estimating some parameters used in system (35), based on the measurements of tumor cells. Our goal is to accurately describe the dynamics of tumor growth on an individual basis which is very important both for growth prediction and designing personalized, optimal therapy schemes (e.g., when using model predictive control). To demonstrate this, let us consider two of the parameters in the model, namely, *c*_1_ which is the competition rate that impacts the dynamics of tumor cells due to natural killer cells and *d*_3_ which is the parameter-related proliferation of dendritic cells due to tumor cells. Recalling the influence of doubling the latter parameter *d*_3_ is illustrated in Figure 3.

**Figure 9:**
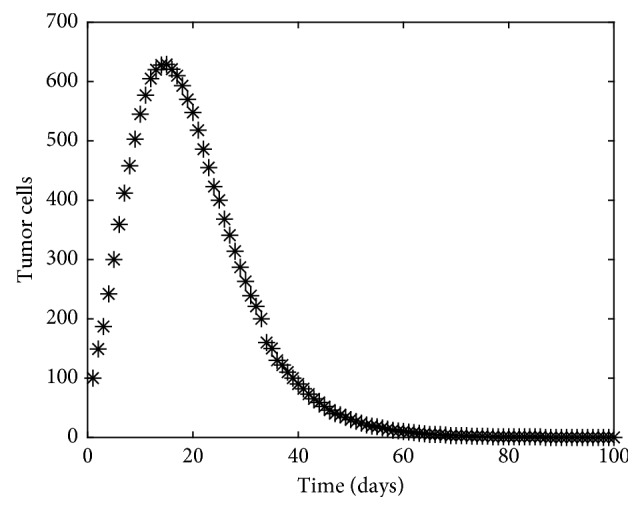
Experimental data for tumor cells for *c*_1_=3.5 × 10^−6^ and *d*_3_=1 × 10^−4^ with some noise.

**Figure 10:**
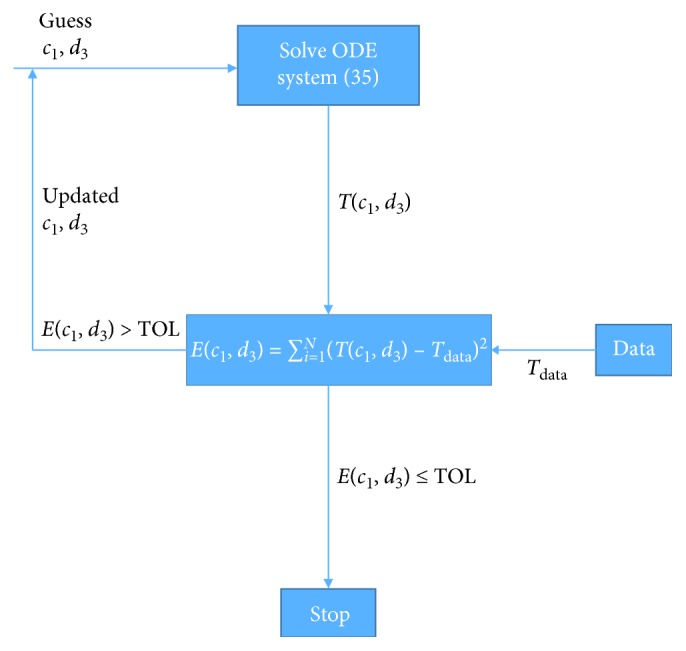
Parameter estimation description.

The purpose of parameter estimation is to identify values of parameters for given experimental data. In this work, we will demonstrate how to check the reliability of a mathematical model to estimate parameters optimally. For this, we consider a discrete dataset for tumor dynamics corresponding to the values of *c*_1_=3.5 × 10^−6^ and *d*_3_=1 × 10^−4^ as indicated from the literature (see Figure 9). We then introduce randomness in the data by adding some Gaussian noise to each value in the tumor dynamics. We refer to the latter as the experimental data *T*_data_ (see Figure 10).

Next, we make a guess for values of *c*_1_ and *d*_3_ which are very different from the values used to create the experimental data and try to solve the ODE system (35) to obtain the computed values of the tumor dynamics given by *T*(*c*_1_, *d*_3_).

We then set up an error expression *E*(*c*_1_, *d*_3_) that is the sum of the squared differences between the computed values *T*(*c*_1_, *d*_3_) and the experimental data *T*_data_ as shown in equation (36). Employing an unconstrained nonlinear optimization algorithm such as the Nelder–Mead algorithm, the minimization algorithm searches for a local minimum using a regression approach. This direct search method attempts to minimize a function of real variables using only function evaluations without any derivatives. If the error *E*(*c*_1_, *d*_3_) is within a user-prescribed tolerance TOL, we stop and accept the values of *c*_1_ and *d*_3_ to be the optimal values. If not, we use the updated values of parameters *c*_1_ and *d*_3_ as the new values and iterate them back to solve the ODE system (35). We continue this until convergence. This parameter estimation approach is summarized in Figure 10, and the minimized objective function is given by

(36) $$\begin{array}{c} E\left(c_{1},d_{3}\right)={\overset{N}{\underset{i-1}{\sum }}\left(T\left(c_{1},d_{3}\right)-T_{\text{data}}\right)}^{2}, \end{array}$$

where *E*(*c*_1_, *d*_3_), the least squared error, denotes the differences in the amount of computed tumor cells from the simulation *T*(*c*_1_, *d*_3_) from the observed data *T*_data_ (Figure 9) over *N* observations.

**Figure 11:**
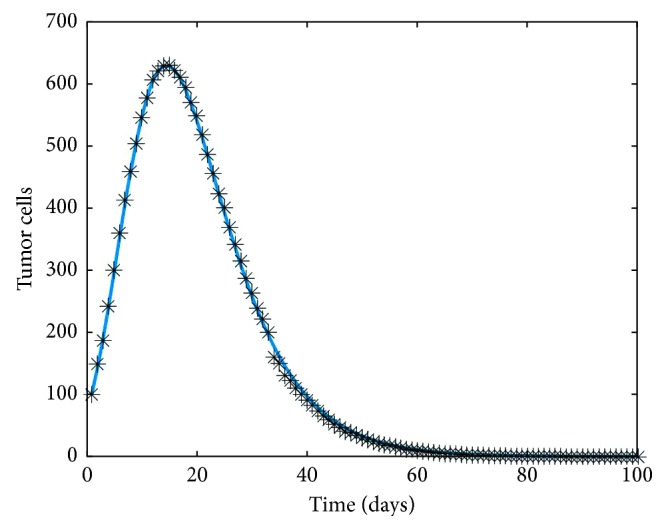
Prediction of the dynamics of tumor cells through estimated values for *c*_1_=3.5 × 10^−6^ and *d*_3_=0.45 × 10^−3^ in solid line plotted with the data.

Using same initial conditions with poor guesses for *c*_1_=1 × 10^−8^ and *d*_3_=1 × 10^−2^, the optimization algorithm estimates the parameters to be *c*_1_=3.5 × 10^−6^ and *d*_3_=0.45 × 10^−3^. Clearly, the algorithm estimates the values pretty close to the values used originally to create the experimental dataset, and the predicted dynamics of the tumor cells for these parameter values along with comparison to the experimental data is illustrated in Figure 11.

## 6. Discussion and Conclusion

In this work, we developed a mathematical model that incorporated the dynamics of four coupled cell populations including tumor cells, natural killer cells, dendritic cells, and cytotoxic CD8^+^ T cells that influence the growth of tumors. The novelty in the model was how it combined important interactions between growing tumor cells and cells of the innate and specific immune system coupled with models for drug delivery to these cell sites. A detailed stability analysis of the associate ordinary differential equation system was performed. A variety of computational experiments were simulated to study the influence of the dynamics of the four cell populations on various parameters. For example, we noted a significant effect of the influence of proliferation rate *d*_3_ on the dynamics of the cell populations which was neglected in past studies. We noted that the dynamics are a function of the source terms included in the model. The effect of TIL drug intervention as an immunotherapy approach had significant impact on tumor growth. Similar results were observed for a chemotherapy approach as well. Clearly, a combined chemotherapy and immunotherapy drug intervention approach was seen to reduce tumor growth greatly. Another feature of the work is the application of a parameter estimation algorithm to accurately predict proliferation parameters for a given set of data of tumor cell growth. Similar algorithms have been used in the past to characterize properties of soft tissues [40]. We were not only able to capture the data accurately but also were able to accurately quantify the parameters related to the data.

While this paper investigates a system of ordinary differential equations, one must computationally study the corresponding partial differential equations (PDES) presented as we developed the model along with fluid equations that help the drugs to move towards the cancer cells. This will require the use of sophisticated numerical methods like the finite element methods to solve the associated system of PDEs. This will be the focus of a forthcoming paper.

Also, we hope to extend our work to apply the models and validate them against actual experimental or laboratory data along with applying machine learning type algorithms to predict behaviors of the growth of the tumor cells. These predictions can help develop control mechanisms such as drug therapy. This will also be a focus of our future work.

## Data Availability

All data supporting the results reported have sources provided in the manuscript through references or generated during the study.
